# *Metarhizium anisopliae*: A fungal biocontrol agent against *Rhipicephalus microplus*

**DOI:** 10.1371/journal.ppat.1013918

**Published:** 2026-01-30

**Authors:** Júlia de Fraga Sant’Ana, Lucélia Santi, Walter Orlando Beys-da-Silva

**Affiliations:** 1 Faculty of Pharmacy, Federal University of Rio Grande do Sul, Porto Alegre, Brazil; 2 Center of Biotechnology, Federal University of Rio Grande do Sul, Porto Alegre, Brazil; University of Maryland, Baltimore, UNITED STATES OF AMERICA

## Parasitism of *Rhipicephalus microplus* and chemical control

The parasitism activity of the cattle tick *Rhipicephalus microplus* is responsible for direct and indirect impacts on cattle: blood loss, anemia, decreased milk production, predisposition to myiasis, leather damage, weight loss, udder damage, abortions and death, and the transmission of pathogens that can cause theileriosis, babesiosis, anaplasmosis, and intraerythrocytic rickettsia [1–3]. These effects lead to worldwide losses estimated at over US$13.9 billion per year [3].

To mitigate the significant economic impact, the most commonly used strategy to control the *R. microplus* is through chemical acaricides. However, the use of chemical compounds poses serious concerns, including environmental pollution, risks to human and environmental toxicity [4,5] and to the selection of resistant strains from the cattle tick. A study with field samples of *R. microplus*, from Rio Grande do Sul state (Brazil), in the presence of chemical acaricides found that 78.85% of them presented resistance to three or more compounds [6]. In Benin, samples of the cattle tick were collected from five farms in four agro-ecological zones, where three chemical acaricides were popularly used, finding only one of the five populations was susceptible to alpha-cypermethrin while the other four were resistant, from which two showed an even higher resistance to all acaricides [7]. These findings are especially concerning given the nearly worldwide distribution of *R. microplus*—covering most of South America, across Central America, south of North America, sub-Saharan Africa, Western Europe, Southeast Asia, and coastal parts of Australia. Predictive models indicate that climate change may further expand its range into neighboring regions currently considered unsuitable, exacerbating the problem [8].

**Fig 1:**
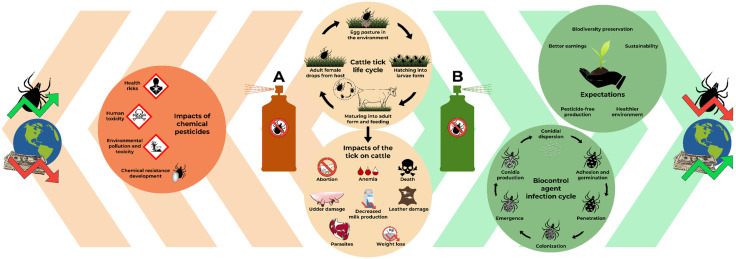
Cattle tick’s life cycle, its impacts on cattle, and how chemical (A) or biological (B) control can interfere in this scenario. In A: human toxicity, health danger, environmental pollution, toxicity, and chemical resistance are expected, leading to a continuous economic hazard due to chemical pesticide use to control the cattle tick. While in B: after the biocontrol agent fulfills its infection cycle in the cattle tick, a healthier environment preserving the biodiversity is expected than that with the use of chemical acaricides, giving producers better earnings and pesticide-free productions, leading to a sustainable economic result, declining the cattle tick infestation.

To avoid the chemical resistance, non-acaricidal strategies can be adopted for the control of the cattle tick, such as the release of sterile male hybrid ticks, grazing management of pasture, and biological control, which can be defined as the use of one live organism for the reduction of the population of another organism, such as pests or pathogens. Among the examples of control agents are natural predators, pathogens, and parasites [9]. Besides contributing to dodging the chemical resistance led by pesticides, biocontrol is also a more sustainable alternative, which promotes a healthier environment, pesticide-free products, and better earnings for producers, preserving local biodiversity [10]. In this work, the qualities and limitations of cattle tick biocontrol using an entomopathogenic and acaricide fungus will be addressed, along with molecular insights regarding its virulence and pathogenicity. In Fig 1, the cattle tick’s life cycle is compiled with its effects on cattle, and the impacts of using chemical or biological control, given the environmental and economic implications.

## Biocontrol with entomopathogenic and acaricide fungi

Pathogens, such as entomopathogenic and acaricide fungi, can be used for the biocontrol of the cattle tick, as they are the most used in biocontrolling arthropod pests [11]. Fungi from the genus *Beauveria*, *Isaria*, and *Metarhizium* can be cited as promising examples of environment-friendly biocontrol agents, since they have been developed into extensively applied mycoinsecticides and mycoacaricides, although there is limited data about the market of biocontrol agents produced by smaller companies [12].

Regarding the use of *Metarhizium anisopliae* as a biocontrol agent, *in vitro* bioassays using *R. microplus* engorged females demonstrated the potential use of this fungal species to infect and to control the cattle tick for the first time, selecting the E6 strain as the most promising one among several isolates [13]. A pioneer trial was also conducted using *Beauveria bassiana* and *M. anisopliae*, demonstrating their virulence towards *R. microplus* cuticle and eggs, respectively [14]. This study also evaluated the action of *M. anisopliae* in the field, through spraying infested animals with the suspension, finding significant alterations for indices used to assess reproductive and nutritional efficiencies. Another field study compared the efficacy in the control of the cattle tick by the application of only chemical acaricides, only *M. anisopliae* conidia suspension, or a combination of both, and found statistically similar rates for their separate uses and a higher rate for their combined use [15]. The exposure of different tick species (*Rhipicephalus annulatus*, *Hyalomma excavatum*, and *Rhipicephalus sanguineus*) to several strains of different acaricide fungi, resulted in a display of higher virulence of *M. anisopliae* strains when applied on engorged *R. annulatus* and *R. sanguineus* females and to all eggs and unfed larvae, therefore showing its potential for the biocontrol of those tick species as well [16].

Despite these promising results, the biocontrol of cattle ticks using *M. anisopliae* remains limited by the environmental stresses that conidia must endure under field conditions. When applied to livestock in pastures, paddocks, or pens, the fungus may suffer from ultraviolet radiations, temperature variation, and low humidity, which can limit the effectiveness and application possibilities of the formulation by impacting the conidia to a molecular level [17]. Another important limitation to the use of microbial agents is the time required for the effective pest control, which is longer than what is needed for conventional pesticides [18].

## Molecular insights on tick biocontrol with *M. anisopliae*

To better comprehend the mechanism of action of *M. anisopliae* as a biocontrol agent, proteomic studies have shown promise, as they reveal molecular players and processes involved in the infection and are pivotal to the effectiveness of biocontrol action. The proteomic studies results using distinct insects (*Bombyx mori*, *Callosobruchus maculatus*, *Coptotermes curvignathus*, *Dermolepida albohirtum*, *Dysdercus peruvianus*, *Galleria mellonella*, *Helicoverpa armigera*) and arachnids (*R. microplus*) by different strains of *M. anisopliae*, identify several proteins linked to different steps of infection [19]. One study reviewed a series of proteins differentially expressed by conidia and mycelia of *Metarhizium* spp. when analyzed in the context of biocontrol of different arthropods, which could be recognized in different steps of infection, such as adhesion, penetration, signaling, and fungal defense, therefore performing important tasks in each step [19], as follows.

**Fig 2:**
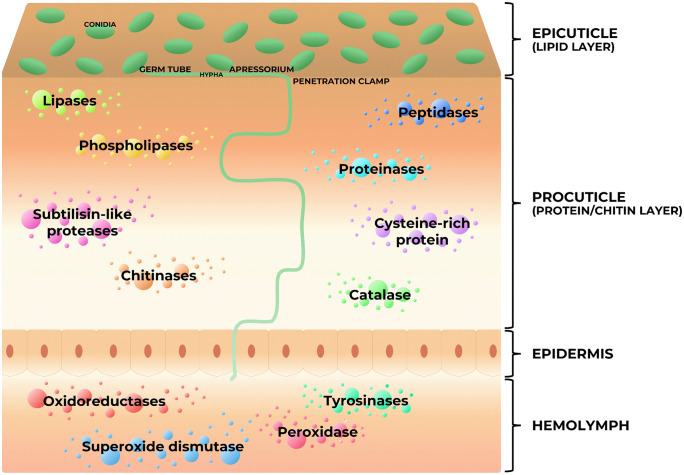
Infection model of the cattle tick by *Metarhizium anisopliae* considering proteins expressed during infection.

Fungal effectors are proteins that exert a key role in infection processes, especially for entomopathogenic fungi, as they can manipulate the host through targeting its immune system [20]. Some examples include oxidoreductases, catalases, peroxidases, superoxide dismutases, cysteine-rich proteins, tyrosinases, chitinases, and subtilisin-like proteases [21]. The activity of these proteins in each step of the infection leads to biocontrol effectiveness, and some of those molecular players are directly linked to the virulence of the isolate [22]. Before adhesion, the fungus recognizes the host through the lipids present in the epicuticle, and then uses lipolytic enzymes to increase hydrophobicity, along with the use of hydrophobins, adhesins, and other proteins, such as GAPDH (gluceraldehyde-3-phosphate dehydrogenase) and phosphatases for adhesion [17]. Afterwards, the conidia germinate, forming the germ tube and then the appressorium, a specialized hypha defined as a swollen hyphal tip that adheres to the host surface, facilitating fungal penetration into the arthropod host. Inside the appressorium, turgor pressure increases, and a fine growing point, called the penetration peg, is formed. Together with enzymes, it results in physical and chemical pressure, respectively, on the host’s cuticle surface. Accordingly, the expression of genes related to those infection structures, mainly hydrolytic enzymes, is triggered, while the host activates its first defense mechanisms. For the cuticle penetration process, the action of lipases, proteases, and chitinases are needed since the host´s cuticle is composed by lipids, proteins, and chitin. These enzymes also help to support fungal growth nutritionally, breaking the polymeric substrates and releasing monomers for fungal development through the different layers of the cuticle during the pathogen’s infection until it reaches the hemolymph [17,23]. Once deeper into the arthropod body, the fungus modulates the host’s immune system to prevail, through the secretion of more fungal effectors, such as oxidoreductases and tyrosinases [19]. Other studies have also found a series of enzymes expressed by *M. anisopliae* in different infection models of the cattle tick [23–25]. Fig 2 proposes a model of their display in cattle tick infection.

Considering the scenario encompassing the previously discussed impacts of *R. microplus* parasitism, its ever-evolving resistance towards chemical acaricides, and the predictions of climate change facilitating its territorial expansion, identifying more effective control strategies is a pressing matter. In this context, biocontrol using entomopathogenic and acaricide fungi, particularly *M. anisopliae*, emerges as a viable alternative, and is also associated with the promotion of a healthier environment. However, to obtain strains or isolates with higher virulence and pathogenicity specific to this host, further studies are required to better characterize molecular mechanisms of infection, as well as to develop improved formulations for field application.
